# Therapeutic drug monitoring of imatinib in paediatric chronic myeloid leukaemia: Data from a real‐world setting

**DOI:** 10.1111/bjh.20047

**Published:** 2025-03-10

**Authors:** Meinolf Suttorp, Stephanie Sembill, Phyllis Lensker, Verena Hildebrand, Elke Schirmer, Axel Karow, Manuela Krumbholz, Manfred Rauh, Markus Metzler

**Affiliations:** ^1^ Pediatric Hemato‐Oncology, Medical Faculty Technical University Dresden Dresden Germany; ^2^ Pediatric Oncology and Hematology, Department of Pediatrics and Adolescent Medicine University Hospital Erlangen Erlangen Germany; ^3^ Comprehensive Cancer Center Erlangen‐EMN (CCC ER‐EMN) Erlangen Germany; ^4^ Bavarian Cancer Research Center (BZKF) Erlangen Germany; ^5^ Pharmacy Department University Hospital Erlangen and Friedrich‐Alexander‐Universität Erlangen‐Nürnberg Erlangen Germany; ^6^ Institute of Experimental and Clinical Pharmacology and Toxicology Friedrich‐Alexander‐Universität Erlangen‐Nürnberg Erlangen Germany

**Keywords:** CML, imatinib, paediatric oncology, pharmacokinetics

## Abstract

Imatinib (IMA) therapy for paediatric chronic myeloid leukemia (pCML) requires age‐dependent dose adjustments. Assessment of therapeutic drug monitoring (TDM) under ‘real‐world’ conditions was performed. Collection of blood and TDM‐relevant data, calculation of individual dosage exposure, measurement of plasma *C*_min_ (IMA, Nor‐IMA) by HPLC/MS‐MS and recording of adverse event (AE). Two hundred and forty‐six specimens from 66 patients were analysed. Individual median IMA dosage exposure was 253 mg/m^2^ (range: 128–504). Children <13 years received a median of 43 mg/m^2^ more than older patients (*p* < 0.0001). Median *C*_min_ of IMA and Nor‐IMA was 1017 ng/mL (range: 51–3976) and 269 ng/mL (range: 21–981), respectively, correlating significantly with the prescribed dose. At 5/246 visits, non‐adherence was confirmed by very low IMA *C*_min_ in 3/66 patients, all ≥13 years old. Correlation of IMA *C*_min_ >1000 ng/mL with achieving OMR demonstrated in each 63% (*N* = 24/37, *N* = 27/43, respectively) patients at months 3 and 6. In the cohort with lower levels, only 23% and 50%, respectively, achieved these milestones. This difference was significant only at month 3. Of 66 patients, 30 reported 125 AEs with gastrointestinal and musculoskeletal as leading complaints. In 9.3% of AEs, the correlated IMA *C*_min_ was ≥3000 ng/mL. TDM is a simple and rapid additional tool for managing pCML under ‘real‐world conditions’.

## Graphical abstract

Therapeutic drug monitoring (TDM) of imatinib is reported from a real‐world study in 66 children with paediatric Chronic Myeloid Leukemia (pCML). TDM allows dose adjustments, improving molecular response rates and is a step forward in personalized pCML care.

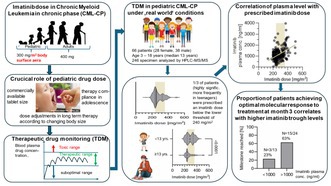

## INTRODUCTION

Targeted treatment with the tyrosine kinase inhibitor (TKI) imatinib (IMA) has impressively improved the probability of survival of chronic myeloid leukaemia (CML).^1^, ^2^ Patients on TKI treatment achieving and maintaining a deep molecular response (DMR) over several years may even stop taking the drug and remain in a state of ‘operational cure’ with >50% probability.^3^, ^4^ To achieve this therapeutic goal, optimal therapy management plays a crucial role.

It was suggested that variations in blood plasma IMA trough levels (*C*_min_) could affect cytogenetic and molecular responses.^5^, ^6^, ^7^ Results of several trials confirmed that a threshold IMA *C*_min_ exposure of >1000 ng/mL is associated with higher incidences of response rates.^7^, ^8^, ^9^ In addition, a relationship between higher IMA concentrations (>3000 ng/mL) and adverse events (AEs) has been described.^7^, ^10^, ^11^ For adult patients with CML, consensus guidelines for therapeutic drug monitoring (TDM) of IMA have been published.^12^

In children, however, the scenario is different. With an age‐related annual incidence of 0.07/100 000 in 1‐ to 14‐year‐old children, pCML is a rare disease contributing 2%–3% to all childhood leukaemias, which makes clinical studies considerably more challenging.^4^, ^13^, ^14^ Existing treatment recommendations for pCML, therefore, overlap to a great extent with recommendations for adult patients^3^, ^15^, ^16^ but it could also be demonstrated that pCML differs from adult CML by biological factors and age‐related side effects of TKI therapy such as growth stunting.^17^, ^18^

Early phase 1/2 pharmacokinetic (PK) trials have investigated IMA levels and its major metabolite norimatinib (Nor‐IMA) in small numbers of children and adolescents.^19^, ^20^, ^21^ While data are lacking in children younger than 3 years, the PK data of IMA in children are generally not significantly different from those in adults.^22^ This should allow the translation of most of the TDM data to pCML; however, formal proof in paediatric cohorts is still missing.^23^

Several practical particularities must be considered when setting the optimum paediatric IMA dosage. The available tablet sizes (smallest size 100 mg, dividable into 50 mg) allow only a coarse approximation of the calculated target dose, especially for smaller children. Given the significant growth and weight gain during childhood, the dose should be adjusted regularly. Adherence to treatment at different ages is another factor exerting a strong influencing factor, all of which are usually only anecdotally reported and hardly quantified.

In a large cohort of children, we here evaluated IMA *C*_min_ and its metabolite Nor‐IMA *C*_min_ in blood samples collected for regular *BCR::ABL1* transcript quantification or IMA *C*_min_ testing due to specific clinical indications. The aim was to determine the extent to which TDM provides guidance for dose optimization in balancing optimal response and minimizing AEs during IMA treatment.

## MATERIALS AND METHODS

Two hundred and sixty‐one blood samples from 66 patients (male: 38, female: 28) diagnosed with CML in chronic phase (CML‐CP) and enrolled in the pCML registry were sent to the study laboratory between 8/2018 and 12/2022. The age at diagnosis ranged from 3 to 17 years with a median of 13 years (see Supporting Information S1). The prospective study was conducted in accordance with the Declaration of Helsinki, approved by the institutional ethics boards (EK282 122 006 and EK 236_18 B), and registered at EUDRACT (2007‐001339‐69) and Clinical‐Trials.gov (NCT00445822). Written informed consent was obtained from the children and/or their legal guardians. Patients received standardized TKI‐based therapy. Data regarding sex, age, clinical and biological features including complications were collected from participating centres at diagnosis and at regular intervals thereafter. Diagnoses were confirmed by central reference review as per the current guidelines of the criteria of the European LeukemiaNet [2] and patients <18 years old diagnosed with pCML from 1/2004 to 12/2022 were eligible for monitoring of plasma IMA *C*_min_.

A 5‐ to 10‐mL EDTA blood was collected at routine clinical visits. The date and time of blood sampling and of the last IMA intake, IMA dose and patient's body weight and height were recorded on the sample submission form. The blood was aliquoted for measuring a full and differential blood count, the *BCR::ABL1* transcript ratio and plasma was separated for determination of IMA *C*_min_ and Nor‐IMA by HPLC coupled with tandem mass spectrometry as described in Supporting Information S5.^24^, ^25^

From the patients' anthropometric data and the prescribed TKI dose, the body surface area (BSA) and the individual IMA dosage (mg/m^2^) were calculated (see Supporting Information S2 for details). For 51 specimens, data were missing. Fifteen of these 51 specimens had to be excluded due to missing essential data. In another 20 specimens, the incomplete anthropometric data were interpolated as the interval to a preceding or following report on these data was shorter than 2 months (for details, see Figure S1B). In the remaining 16 specimens, the time interval from the last drug intake to the collection of blood was ≠24 ± 0.5 h. As the PK profile of IMA is characterized by first‐order elimination, this difficulty can be overcome by calculation of a corrected *C*_min_.^11^, ^19^, ^20^, ^21^, ^26^ Details on the calculation are outlined in Supporting Information S3.^27^ By these means, *N* = 36 *C*_min_ level measurements were re‐added to the cohort. In total, 246 IMA *C*_min_ measurements were analysed in 66 patients ≤18 years old.

Molecular response was assessed by RT‐PCR and data are presented according to the International Scale (IS). Molecular response was categorized according to the recommendations of the European LeukemiaNet defining ‘Optimal Molecular Response (OMR)’ as a BCR::ABL1/ABL1 transcript ratio of ≤10% at month 3, ≤1% at months 6 and ≤0.1% at month 12 respectively.^2^

AEs were recorded at each visit and categorized according to the Common Terminology Criteria for AE (CTCAE) Version 5.0.^28^ The WHO‐UMC system was used for standardized case causality assessment of IMA treatment.^29^ Both categorizations were double‐checked by two clinical pharmacists, and the occurrence of AE was compared with IMA *C*_min_.

Statistical analysis was performed using GraphPad Prism, version 10.2.0 (GraphPad Software, San Diego, California, USA). IMA dose distribution and IMA *C*_min_ levels according to sex and age were compared using the Kolmogorov–Smirnov test. The significance level was defined as *p* < 0.05. Differences in the BCR::ABL1/ABL1 ratio between the low and high IMA plasma concentration groups were tested using the Mann–Whitney test. A two‐tailed *p*‐value of <0.05 was considered statistically significant.

## RESULTS

**FIGURE 1:**
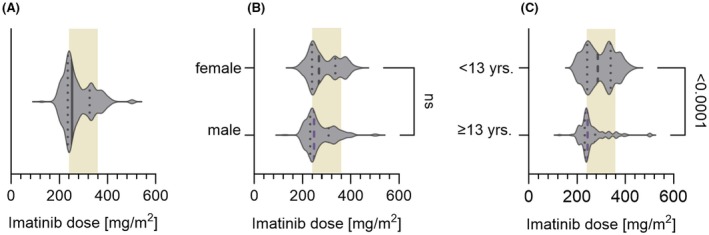
Violin plots showing the individual imatinib dose distribution as administered 24 h before the time point when the blood specimen was collected. (A) In the whole group, the median dose administered (bold line) was 253 mg/m^2^, and the range distribution was 128–504 mg/m^2^. The 25% (235 mg/m^2^) and 75% (326 mg/m^2^) interquartile range is shown by dotted lines. The light brown‐shaded area denotes the recommended dose range (240–360 mg/m^2^) for the treatment of chronic myeloid leukaemia (CML) in chronic phase and accelerated phase (CML‐AP) corresponding to the adult single daily doses of 400 and 600 mg, respectively. (B) Female patients received non‐significantly higher median doses (268 mg/m^2^) than male (248 mg/m^2^) patients. (C) Patients ≥13 years old received a highly significantly (*p* < 0.0001) lower median dose of 243 mg/m^2^ (range 128–502) than patients <13 years old (median dose 286 mg/m^2^ (range 196–429)). Also, the distribution range was broader in younger patients.

Patients received a mean daily IMA dose of 253 mg/m^2^ (range 128–504) (Figure 1A). Of 246 measurements, 82 (34%) stemmed from patients receiving a dose below the recommended lower threshold of 240 mg/m^2^. Girls received a non‐significantly higher median dose (268 mg/m^2^, range: 177–429) than boys (248 mg/m^2^, range: 128–504) (Figure 1B). Separation of the total cohort based on age into two subgroups (≥13 and <13 years old) unravelled significantly higher drug intake in younger children (median dose 286 mg/m^2^, range: 196–429) than in older children (243 mg/m^2^, range: 128–504) (Figure 1C).

**FIGURE 2:**
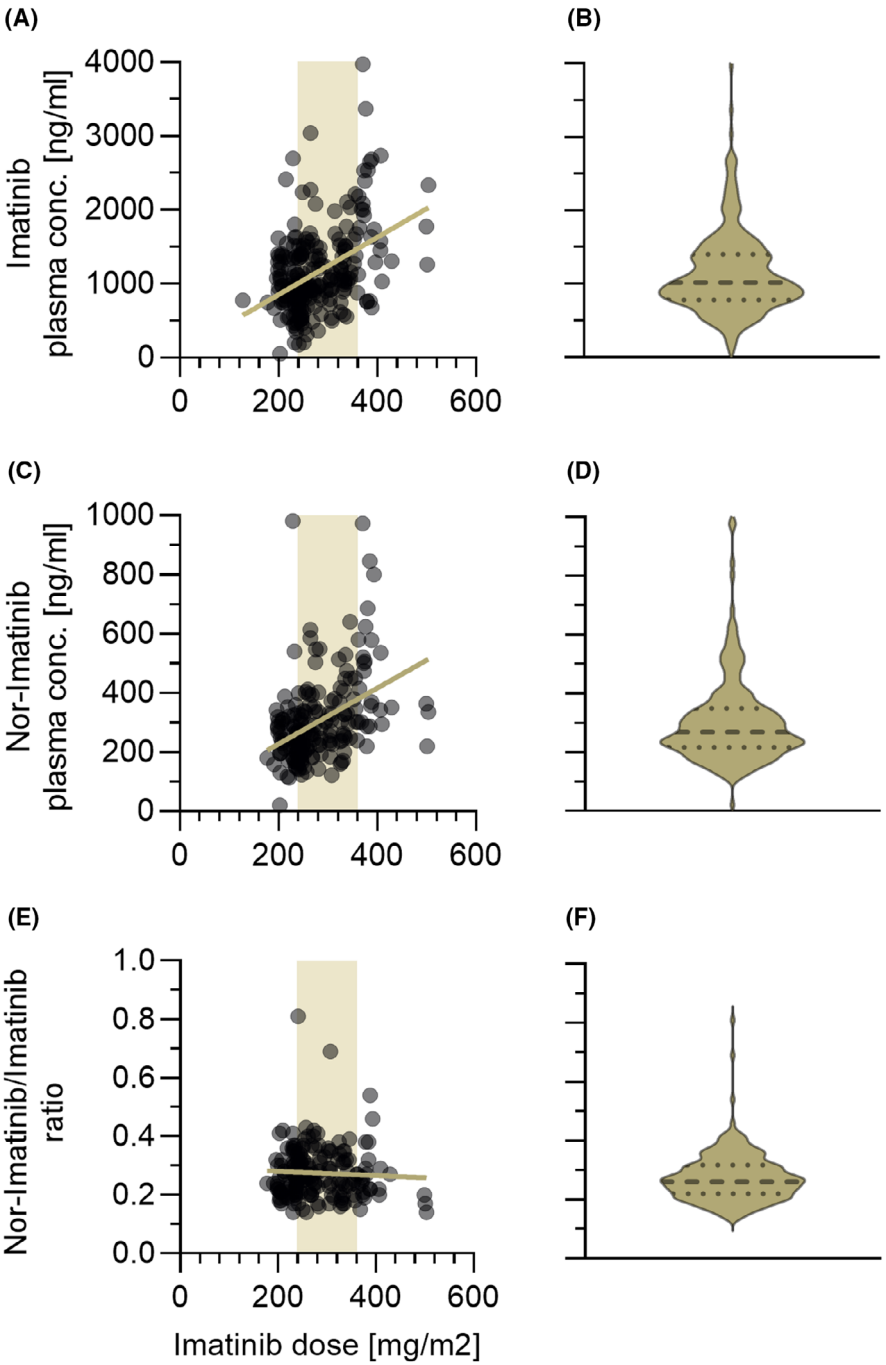
Plasma concentration of imatinib (IMA) (A), its major metabolite Nor‐IMA (C) and the metabolic ratio Nor‐IMA/IMA (E) in correlation to the administered drug dose. Left (A, C, E): Individual measurements are shown as grey dots which may overlay and appear as black areas. The light brown‐shaded area denotes the recommended IMA dose range (240–360 mg/m^2^). The traversing line represents the linear regression. Right (B, D, F): Corresponding violin plots denoting the median (bold‐dashed line) and the 25% and 75% interquartile range (dotted lines).

In the whole cohort, plasma IMA *C*_min_ ranged from 51 to 3976 ng/mL with a median of 1017 ng/mL (dashed line in Figure 2B). Plasma Nor‐IMA levels were measured in the range of 21–981 ng/mL (median: 269 ng/mL) (Figure 2D). Plasma IMA *C*_min_ and Nor‐IMA *C*_min_ levels correlated significantly with the dose of drug intake (Figure 2A,C). The metabolic ratio Nor‐IMA/IMA ranged from 0.14 to 0.81 (median 0.26) and did not correlate with the administered IMA dose (Figure 2E,F).

**FIGURE 3:**
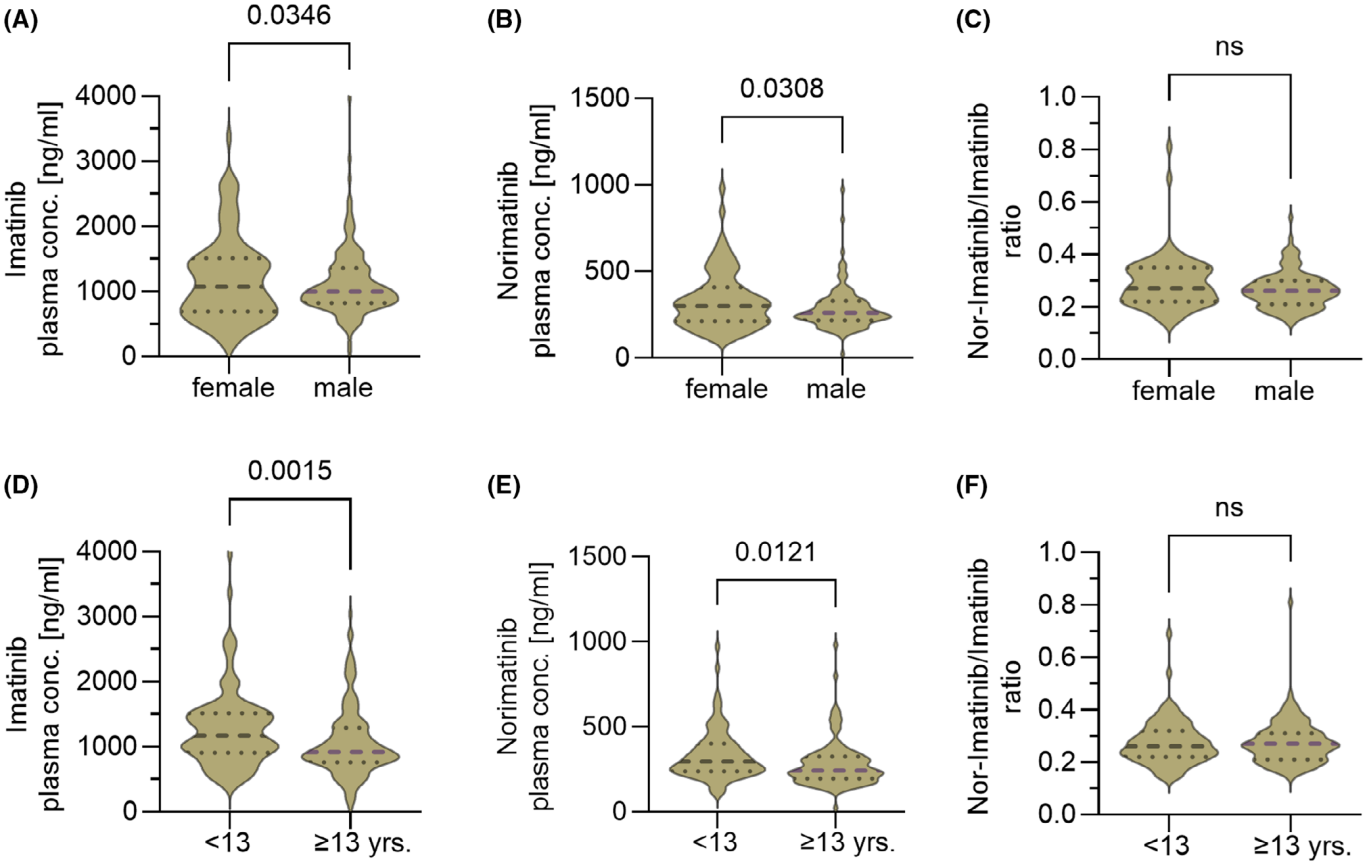
Violin plots illustrate the influence of gender (A, B, C) and age (D, E, F, patients being ≥13 years vs. <13 years) on plasma IMA‐*C*_min_, Nor‐IMA *C*_min_ and the metabolic ratio Nor‐IMA *C*_min_/IMA *C*_min_. (A) Median plasma IMA *C*_min_ is increased in the female cohort (*p* = 0.0346). (B) Median plasma Nor‐IMA *C*_min_ is significantly low (*p* = 0.0308) increased in the female cohort. (C) No significant difference (*p* = 0.0531) with regard to sex is observed in the metabolic ratio Nor‐IMA *C*_min_/IMA *C*_min_. (D) Median plasma IMA *C*_min_ is significantly increased (*p* = 0.0015) in the younger age cohort. (E) Median plasma Nor‐IMA *C*_min_ is increased in the younger cohort (*p* = 0.0121). (F) No significant difference (*p* = 0.06592) was observed in the metabolic ratio Nor‐IMA *C*_min_/IMA *C*_min_ between the two age groups. IMA, imatinib.

When *C*_min_ levels were analysed according to sex, the non‐significantly higher daily drug intake with a broader distribution range in girls (see Figure 1B) translated into a higher median IMA *C*_min_ of 1069 ng/mL (range: 180–3369) compared to boys (median: 996 ng/mL, range: 51–3976) (Figure 3A). In line with this finding, the metabolite Nor‐IMA *C*_min_ in girls was median 299 ng/mL (range: 114–961) and was also higher than in boys (median: 261 ng/mL, range: 21–973) (Figure 3B). The resulting median metabolic ratio Nor‐IMA‐*C*_min_/IMA *C*_min_ did not differ in girls (median: 0.27, range: 0.14–0.81) from boys (median: 0.26, range: 0.14–0.54) (Figure 3C).

Further analysis according to age subgroups (<13 years vs. ≥13 years old) unravelled significantly higher median IMA *C*_min_ in younger children (Figure 3D). In correlation with the highly significantly higher median drug intake in younger children (Figure 1C), the median IMA *C*_min_ was 1174 ng/mL (range: 365–3976) in younger children compared to 919 ng/mL (range: 51–3042) in children ≥13 years old. This also translated into a significantly higher concentration of the metabolite Nor‐IMA *C*_min_ (median: 296 ng/mL, range: 114–973) in younger than in older children (median: 242 ng/mL, range: 21–981) (Figure 3E). The comparison of the metabolic ratio Nor‐IMA‐*C*_min_/IMA *C*_min_ in the two age subcohorts did not differ in younger (median: 0.26, range: 0.14–0.69) from ≥13 years old patients (median: 0.27, range: 0.14–0.81) (Figure 3F).

One hundred and seven and 139 measurements were performed in children <13 years and ≥13 years old respectively. While no IMA *C*_min_ ≤300 ng/mL was detected in the younger cohort, five low‐range measurements were detected in two girls and one boy aged 13, 14 and 17 years respectively. These low levels were observed twice in one girl and one boy (for details, see Supporting Information S4).

Analysis of intra‐ and interindividual IMA *C*_min_ levels over the time of therapy showed a broad variation. No declining or increasing trend of IMA and Nor‐IMA *C*_min_ levels over time was detected, and the metabolic ratio Nor‐IMA *C*_min_/IMA *C*_min_ remained in the same range (for details, see Supporting Information S6).

**FIGURE 4:**
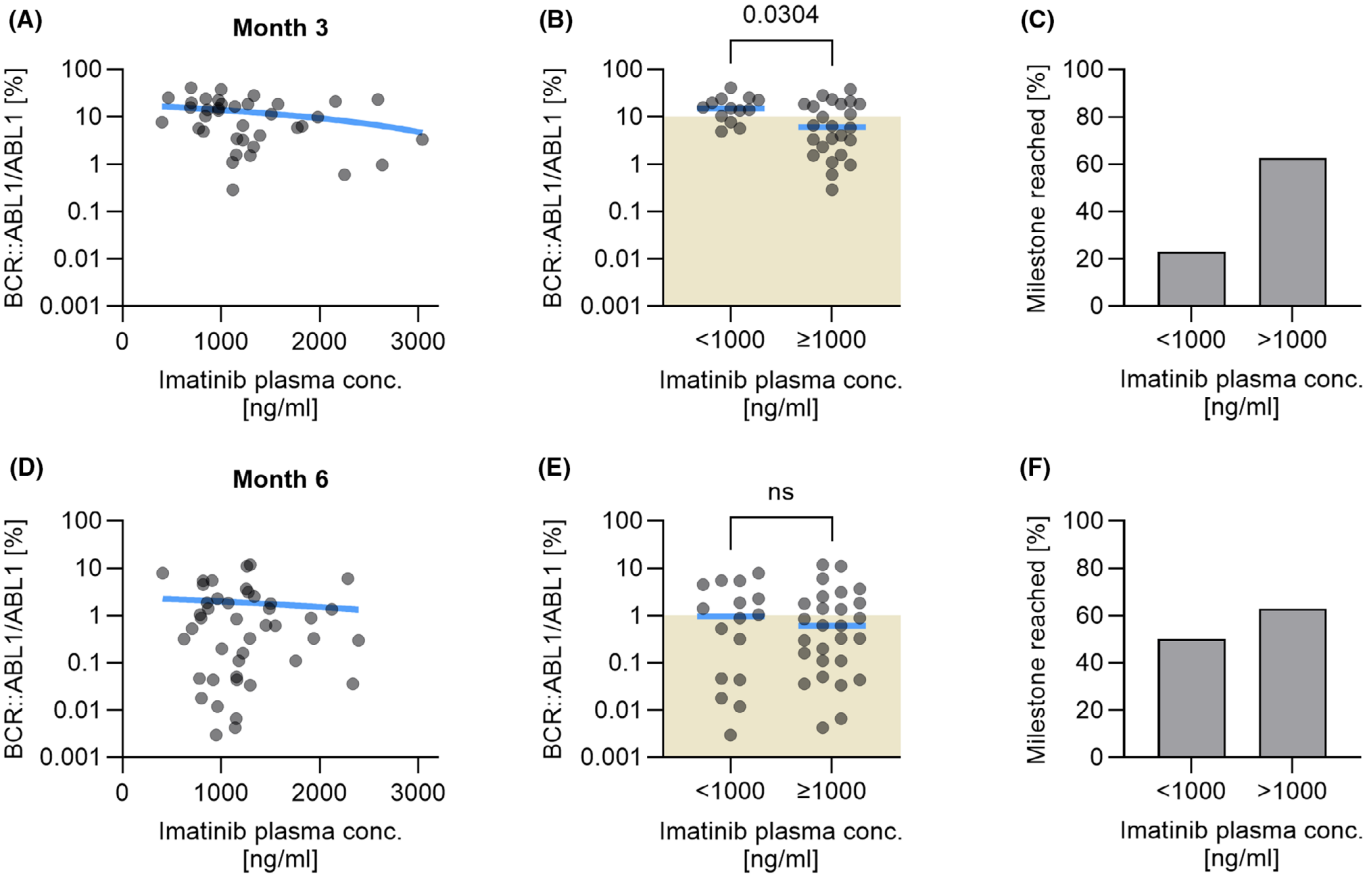
Impact of plasma imatinib *C*_min_ >1000 ng/mL on the achievement of molecular response milestones at month 3 and month 6. (A, D) Individual plasma imatinib *C*_min_ measurements are plotted against the BCR::ABL1/ABL1 transcript ratio in a semilogarithmic fashion at month 3 and month 6 respectively. Individual measurements are shown as grey dots which may overlay and appear as black areas. The blue traversing line denotes the linear regression of a correlation. (B, E) The green‐shaded areas denote the BCR::ABL1/ABL1 transcript ratios indicating an optimal treatment response (10% at month 3, 1% at month 6) as defined by the European LeukemiaNet recommendations. Achievement of optimal treatment response is associated with imatinib plasma *C*_min_ >1000 ng/mL, which reaches a weak niveau of significance at month 3 (*p* = 0.0304) but not at month 6 (*p* = 0.7608). (C, F) At month 3, 63% of patients with plasma imatinib *C*_min_ >1000 ng/mL achieve an optimal treatment response compared to only 23% with lower plasma imatinib *C*_min_. This difference shrinks to 63% and 50%, respectively, at month 6.

To further analyse the impact of IMA *C*_min_ on treatment success as defined by an OMR, IMA *C*_min_ was plotted against the transcript ratio BCR::ABL1/ABL1 at time points month 3 and month 6 after the start of treatment. Linear regression showed a trend but no significant correlation between the transcript ratio and IMA *C*_min_ (Figure 4A,D). For further information, patients were grouped by their level of transcript ratio achieved concerning an OMR as defined by the ELN.^2^

At month 3, an IMA *C*_min_ of <1000 ng/mL was measured in 13/37 patients, and 3 (23%) of these patients achieved an OMR. In the remaining 24 patients, a plasma IMA *C*_min_ of >1000 ng/mL was measured, and 15 (63%) of these patients achieved an OMR. This difference was tested significantly (*p* = 0.0304) (Figure 4B,C). At month 6, an IMA *C*_min_ of <1000 ng/mL was measured in 16/43 patients, and 8 (50%) of these patients achieved an OMR. In the remaining 27 patients, an IMA *C*_min_ of >1000 ng/mL was measured, and 17 (63%) of these patients achieved an OMR, indicating no significant difference (*p* = 0.760) (Figure 4E,F). Further analysis at time point month 12 also showed no correlation of a plasma IMA *C*_min_ higher or lower than 1000 ng/mL with the achievement of an OMR (data not shown).

During the 48‐month study period, a total of 125 AEs were reported by 30/66 patients (1.9 per patient). Details are shown in Table S1. Most frequently, gastrointestinal (32/125; 25.6%) and musculoskeletal/connective tissue disorders (28/125; 22.4%) were reported. For 88/125 (70.4%) side effects, the case causality to the IMA treatment was classified as ‘certain’ or ‘probable/likely’. At the time of measurement, a causality with the IMA *C*_min_ could be predicted for a number of 43 IMA‐related AEs. In a minority of 4/43 (9.3%) side effects, the correlated IMA *C*_min_ was ≥3000 ng/mL.

## DISCUSSION

Here, we report the results of a ‘real‐world’ observational TDM analysis for IMA and its major active metabolite Nor‐IMA in pCML. This is, to the best of our knowledge, the largest analysis in pCML.

In clinical routine outpatient settings, the specific time point to determine a *C*_min_ causes some logistical difficulties, especially in a multicentre approach. Appointments for monitoring outpatients are planned during the day. Most of the children were advised to take IMA with the evening meal to oversleep potential side effects.^17^ Thus, blood collection for TDM exactly 24 h after the last tablet intake (‘true *C*_min_’) is difficult to organize in an outpatient setting, and a true *C*_min_ is almost impossible to measure.^6^, ^20^, ^30^, ^31^ This challenge could be overcome by estimating the *C*_min_ from a sample taken within an interval of ±6 h away from a true *C*_min_.^27^ The observed broad inter‐ and intraindividual variability of IMA *C*_min_ questions efforts to determine the values with scrupulosity. Also, the recent TDM consensus guideline for IMA states that it is important to use an extrapolation of observed values to relate to *C*_min_ reference values.^12^, ^32^

Paediatric recommended IMA doses are BSA‐based and range from 240 to 300 mg/m^2^ in CML‐CP^13^, ^19^ providing systemic exposures similar to 400 mg in adults. Doses >400 mg may yield improved responses; however, they are associated with greater toxicity.^33^, ^34^ From population‐based PK studies in adults, there is no evidence that the patient's size affects the IMA *C*_min_.^26^, ^34^ However, studies in adult Japanese patients found that BSA exerted an effect as 300 mg/day IMA sufficed to achieve an optimal response for patients with small body size.^35^ In paediatric PK studies, total body weight was the only covariate found to significantly affect the plasma IMA clearance and distribution volume.^21^

IMA *C*_min_ in our whole cohort covered a broad range and correlated linearly to the administered dose. These findings confirm earlier PK and population‐based PK studies in children^19^, ^36^ suggesting an optimal dosing regimen range of 240–300 mg/m^2^ daily for CML‐CP. TDM has only spuriously been performed in larger pCML cohorts. In a paediatric study not indicating the individual given dose precisely (‘regular dose’) and the blood collection time (‘at the end of the dosing interval’), 31 patients exhibited a median IMA *C*_min_ of 1820 ng/mL which is higher than in our cohort.^31^

The administered daily mean IMA dose of 253 mg/m^2^ is in the lower recommended range. However, the distribution of doses was skewed, with a 33% proportion receiving less than the recommended lowest dose of 240 mg/m^2^ (Figure 1). As only tablets with a minimal drug content of 100 mg (dividable into 50 mg single dose) were available, this may in part be due either to a prescribing physician's fear of overdosing when rounding up to the next 50 mg increment or a missed dose adjustment when the body weight had increased over the time of treatment. It must be remembered that IMA exposure in children exerts an impact on longitudinal growth, resulting in stunting.^18^, ^37^ This underlines the importance of using updated anthropometric data for BSA calculation and to judge the correct paediatric IMA dosage. Patients aged ≥13 years received a significantly lower median IMA dose than younger children (deficit 14%–18% of the recommended dose range) (Figure 1C). The underlying reason remains speculative: probably splitting a 100‐mg tablet was avoided in teenagers, and instead of a more appropriate 350 mg dose (3½ tablets), only 300 mg (3 tablets) was prescribed.

Earlier studies in adults^6^, ^38^, ^39^ and a recent randomized study with an enormous number of >2400 CML patients identified male, young individuals having low IMA *C*_min_ and lower response rates.^11^ However, in one clinical outcomes trial, women have been described to have a poorer prognosis^1^ while no such sex differences have been observed by others.^40^ In a paediatric cohort with 31 patients, no significant influence of sex and age on IMA *C*_min_ was observed^31^ while in our cohort, the median IMA *C*_min_ was higher in girls than in boys. However, this difference must be interpreted on the background that also the median IMA dose girls received was 20 mg/m^2^ higher than in boys.

Whether or not poorer compliance in teenagers when compared to childhood age contributed to the lower *C*_min_ at teenage age can only be speculated. *C*_min_ measurements <300 ng/mL can be taken as proof that IMA was not taken within the last 24–48 h. This was observed in three patients ≥13 years old but not in younger children. It is suggestive to speculate that, in younger children, the caregivers are administering the tablets while, in teenagers, the patient's desire for autonomy negatively impacts adherence. The phenomenon of poor adherence to TKI intake in pCML has been described before^17^, ^41^ and is a well‐known hurdle to outpatient oral medication regimens also in other paediatric malignancies.^42^, ^43^

When analysed for the first time in a small series of four children, the half‐life of the metabolite Nor‐IMA was found to be shorter than the parent drug.^44^ These data differ from what is reported in adults where Nor‐IMA shows PK comparable to IMA.^40^ Our data demonstrated a typically lower Nor‐IMA *C*_min_ (median 269 ng/mL) than IMA *C*_min_ being well in the range of paediatric^22^ and adult data.^6^, ^39^ No influence of the dose taken on the metabolic ratio Nor‐IMA/IMA was observed (Figure 2).

As in adults, paediatric intra‐ and inter‐patient IMA *C*_min_ varies considerably with a coefficient of variation of 40%–60%.^45^ This can only be partially explained by variations in exposure to IMA at the recommended dose range. Cellular uptake and excretion of IMA are mediated by several intra‐ and extracellular transporters on a genetic background. Also, differences in plasma protein binding, especially by alpha 1 acid glycoprotein, may play a role.^30^, ^45^, ^46^ However, it must be acknowledged that these findings in adults have not been regarded as sufficient to recommend specific dose adjustment of IMA and that the underlying causes of the PK variability are not fully understood.

In adults with CML, four seminal studies have examined the relationship between IMA *C*_min_ and the achievement of treatment response. Consistently, it was demonstrated that the mean IMA *C*_min_ levels were significantly higher in patients with a complete cytogenetic response (CCyR) or major molecular response (MMR) than in those without.^5^, ^6^, ^7^ On the contrary, a study with 78 adults did not find such correlation.^47^ However, this study was hampered by a small and biased selection of patients and heterogeneous sampling times.^45^ According to some authors, adherence to the standard IMA dose, rather than dose adjustment based on its *C*_min_, is critical to achieving a DMR^48^, ^49^ while contrarily a large analysis in >2400 adult patients showed a better plasma concentration–effect than IMA dose–effect relationship.^11^

With the limitation that the number of our analysed cases is small, we compared the mean IMA *C*_min_ in patients with OMR according to the ELN milestones with those of non‐OMR. At month 3, patients with IMA *C*_min_ >1000 ng/mL achieved the milestone of OMR significantly more frequently than those with lower IMA *C*_min_. However, no significant differences were found thereafter. Thus, in a strict sense, the impact of maintaining a level >1000 ng/mL in paediatric patients over a prolonged period could not be clearly defined showing that this threshold should be accepted as a lower target range.

Gastrointestinal and musculoskeletal/connective tissue disorders as the most frequently reported AEs (Table S1) are well‐described side effects of IMA.^50^ Our data do not indicate a correlation between higher IMA *C*_min_ and the occurrence of AE, as IMA *C*_min_ was ≥3000 ng/mL in only a minority of 4/43 cases. However, these results should be confirmed in further studies, as we were not able to calculate the IMA *C*_min_ for each reported AE and classify their severity due to a lack of data.

The impact of any therapeutic decision based on *C*_min_ has not been studied in a standardized fashion in pCML under ‘real‐world’ circumstances. Overall, we were able to confirm that the administration of a median IMA dose of 253 mg/m^2^ resulted in a median IMA *C*_min_ of 1017 ng/mL. No data exist for children on the value of serial measurement of TDM over a prolonged period, the correlation of DMR with IMA *C*_min_, the influence of higher doses on the IMA *C*_min_ and the impact of a dosing change on IMA *C*_min_. While individuals with very low or unmeasurable IMA *C*_min_ are easily identified as non‐adherent patients, all other *C*_min_ should be interpreted with caution and only in the context of all the clinical information available.^49^

## AUTHOR CONTRIBUTIONS

*Conception and design*: M. Metzler, M. Suttorp. *Development of methodology*: V. Hildebrand, P. Lensker, M. Metzler, M. Rauh, M. Suttorp. *Acquisition of data (acquired and managed patients, provided facilities*, etc.*)*: M. Suttorp, S. Sembill, V. Hildebrand, A. Karow, M. Rauh, M. Metzler. *Analysis and interpretation of data (*e.g. *statistical analysis, biostatistics, computational analysis)*: S. Sembill, V. Hildebrand, P. Lensker, E. Schirmer, A. Karow, M. Rauh, M. Metzler. *Writing, review, and/or revision of the manuscript*: M. Suttorp, S. Sembill, P. Lensker, V. Hildebrand, E. Schirmer, A. Karow, M. Kumbholz, M. Rauh, M. Metzler. *Administrative, technical or material support (*i.e. *reporting or organizing data, constructing databases)*: M. Suttorp, P. Lensker, V. Hildebrand, E. Schirmer, M. Rauh, A. Karow, M. Metzler. *Study supervision*: M. Suttorp, M. Metzler.

## CONFLICT OF INTEREST STATEMENT

M. Metzler reports received honoraria for participation on advisory boards for BMS, Novartis and Gilead. M. Suttorp reports receiving honoraria for participation on advisory boards for BMS and Novartis. No potential conflicts of interest were disclosed by the other authors.

## Supporting information


Data S1.

## Data Availability

For original data, please contact Markus Metzler at Pediatric Oncology and Hematology, Department of Pediatrics and Adolescent Medicine, University Hospital Erlangen, Erlangen, Germany.
