# Supplementary material for: Therapeutic drug monitoring of imatinib in paediatric chronic myeloid leukaemia: Data from a real‐world setting
Source: Br J Haematol. 2025 Mar 10;206(5):1397–405. doi: 10.1111/bjh.20047 (PMC12078881; doi:10.1111/bjh.20047)
Supplement: Supplementary file 1 — Data S1. [file BJH-206-1397-s001.docx]

**Supplements**

**Supplement 1**

For 51 specimens collected essential data were not documented. 15 of these 51 specimens had to be excluded as a whole because of completely missing essential data. In another 20 specimens, the missing anthropometric data (height, weight) could be interpolated as the interval to a preceding or following report on these data was shorter than 2 months. In the remaining 16 specimens the time interval from the last drug intake to the collection of the blood specimen was longer or shorter than 24 ± 0.5 hrs. Here a corrected trough level could be calculated as outlined in the section Supplement 2. By these means, N= 36 interpretable trough level measurements were readded to the cohort (Fig. S1a). Thus, 246 valid and interpretable IMA trough level measurements were analyzed in 66 patients <=18 years old.


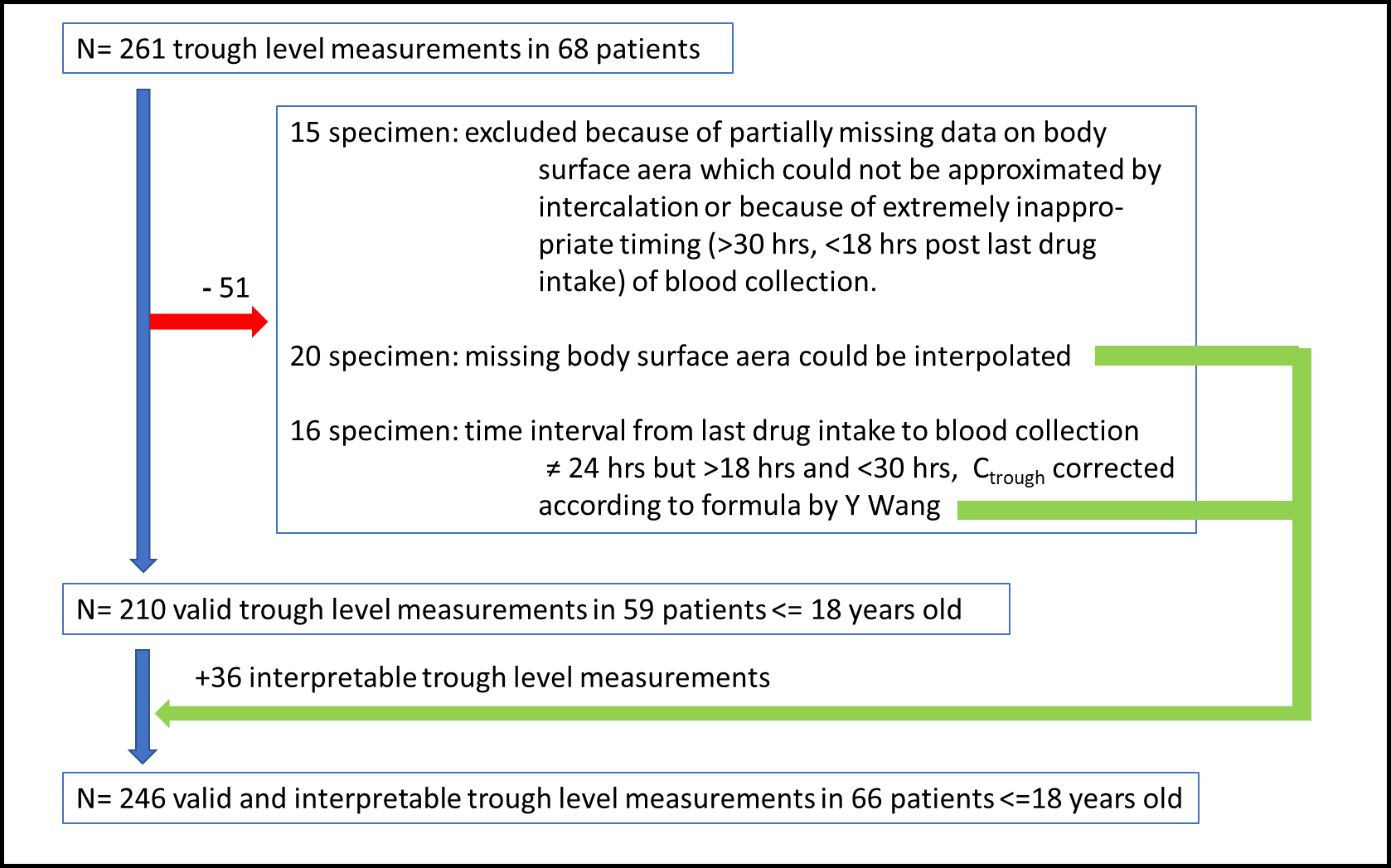


**Figure S1a**: Consort diagram on the number of patients and reasons for inclusion and exclusion of specimen

**Age distribution of the cohort**

The age distribution (range 3 – 17 years) of the cohort was typical for pCML (Fig. S1b). No case younger than 3 years old was observed. Median age was 13 years confirming older observations that the incidence of pCML is rarer in the first compared to the second decade of life.


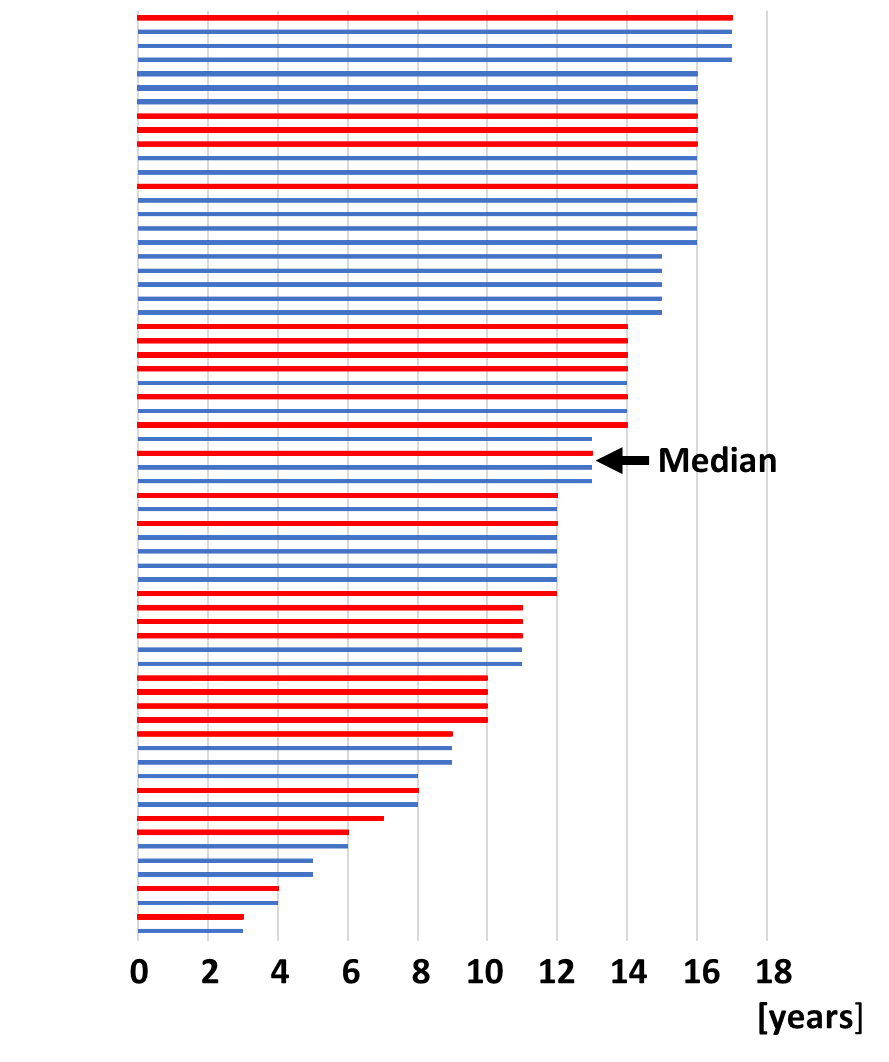


**Figure S1b:** Age distribution of the 66 patients at diagnosis. Blue bars denote boys (N= 38)

and red bars denote girls (N= 28).

**Supplement 2**

**Calculation of body surface area and individual IMA dosage**

The body surface area (BSA) in m^2^ was calculated according to the formula of R.D. Mosteller [1]

$$\text{BSA [m²]} = \sqrt{\frac{\mathrm{height}\left[ \mathrm{cm} \right]\times weight [kg]}{3600}}$$

By dividing the amount of prescribed TKI dose by the calculated BSA the individual IMA dosage (mg/m^2^) was calculated.

Patients’ body mass index (BMI) was calculated according to the formula:

BMI = body length [m] / (body weight [kg])^2^

and interpreted based on age-matched BMI percentiles [2].

**References Supplement 2**

1 Mosteller RD.

Simplified calculation of body-surface area.

N Engl J Med. 1987 Oct 22;317(17):1098. doi: 10.1056/NEJM198710223171717.

2 www.who.int/toolkits/child-growth-standards/standards/body-mass-index-for-age-bmi-for-age; access 15 April 2023

**Supplement 3**

**Calculation of plasma C_min_ in samples collected not precisely 24 hours after the last intake of imatinib**

The pharmacokinetic profile of IMA in children is characterized by first-order elimination. On this basis, the difficulty can be overcome that not all blood samples were collected precisely at a 24 ± 0.5-hour time interval after the last drug intake. As described elsewhere, the drug’s concentration during the elimination phase at a given time point (t_2_) can be calculated from the concentration measured at another time point (t_1_) based on the following pharmacokinetic equation: [1, 2]

Ct_2_ = Ct_1_ x *e* ^(−kE^ ^× ∆t)^

In this equation, ∆t = t_2_ – 24 represents the difference between the moment of blood withdrawal (t_2_ hours after the last drug intake) and the time point (24 hours) for the determination of an ideal C_min_ if the drug is taken once daily. We allowed a blood sampling window extended to +/-6 hours from the time point of 24 hours after the last intake of IMA. As suggested by other authors in children with a bodyweight >40 kg we used for the elimination constant k_E_ the typical adult value of 0.041 per hour, thus reducing the percent deviation at 6 hours to 10.2%, 0%, and 19.0% for patients with low, typical, and high k_E_ values, respectively [1, 2].

By filling in this k_E_ -value the equation reads: Ct_2_ = Ct_1_ x *e* ^(0.041 × ∆t)^. For the chosen limits of ∆t ranging from 18 – 24 = -6 hours (too early) up to 30 – 24= +6 hours (too late), the multiplicator was in the range of e^-0.24^ to e^+0.24^ and the resulting calculated correction factor in the range of 0.78 to 1.27. By multiplication of the measured value Ct_1_ with each correction factor the product Ct_2_ was calculated, thus representing the estimated C_min_.

In children weighing less than 40 kg the k_E_ -value was calculated based on data derived from a pediatric population pharmacokinetic model showing that the pharmacokinetics of IMA and Nor-IMA were well described by one and two-compartment models, respectively, with total body weight being the only covariate found to significantly affect IMA clearance (CL) and distribution volume (V) [3, 4]. By applying the clinically observed pediatric values for CL [L/hr] and V [L] as described by the authors, the influence of body weight on CL is expressed as CL= 10.8 x (body weight [kg] / 70)^0.75^ and on the distribution volume as V = 284 x (body weight [kg] / 70) resulting in k_E_ = CL / V = 0.038 x (body weight [kg] / 70) ^-0.25^.

**References Supplement 3**

1 Wang Y, Chia YL, Nedelman J, Schran H, Mahon FX, Molimard M.

A therapeutic drug monitoring algorithm for refining the imatinib trough level obtained at different sampling times.

Ther Drug Monit. 2009 Oct;31(5):579-84. doi: 10.1097/FTD.0b013e3181b2c8cf.

2 van Eerden RAG, Oomen-de Hoop E, Noordam A, Mathijssen RHJ, Koolen SLW.

Feasibility of Extrapolating Randomly Taken Plasma Samples to Trough Levels for Therapeutic Drug Monitoring Purposes of Small Molecule Kinase Inhibitors.

Pharmaceuticals (Basel). 2021 Feb 4;14(2):119. doi: 10.3390/ph14020119.

3 Menon-Andersen D, Mondick JT, Jayaraman B, Thompson PA, Blaney SM, Bernstein M, Bond M, Champagne M, Fossler MJ, Barrett JS.

Population pharmacokinetics of imatinib mesylate and its metabolite in children and young adults.

Cancer Chemother Pharmacol. 2009 Jan;63(2):229-38. doi: 10.1007/s00280-008-0730-x.

4 Adiwidjaja J, Boddy AV, McLachlan AJ.

Implementation of a Physiologically Based Pharmacokinetic Modeling Approach to Guide Optimal Dosing Regimens for Imatinib and Potential Drug Interactions in Paediatrics.

Front Pharmocol. 2020 Jan 30;10:1672.

**Supplement 4**

Table S4: Reported adverse events and correlation to the Imatinib therapy and plasma IMAC_min_.

|  | | Number (%) | |
| --- | --- | --- | --- |
| Adverse events | | 125 | |
| Patients with adverse events | | 30 | |
| Causality assessment | |  | |
| *Certain + Probable / Likely* | | *88 (70.4)* | |
| *Possible + Unlikely* | | *37 (29.6)* | |
| Trough levels at time of occurrence  *(Certain + Probably / Likely)* | | 43 (34.4) | |
| *Trough level ≥ 3000 ng/ml* | | 4 (9.3) | |
| *Trough level ≥ 2000 ng/ml* | | 4 (9.3) | |
| *Trough level > 1000 ng/ml* | | 29 (67.4) | |
| *Trough level ≤ 1000 ng/ml* | | 14 (32.6) | |
|  | | | |
| Adverse event categorization | | | |
| Adverse events | | Causality assessment [2] | |
| CTCAE category [1] | Number (%) | Certain + Probable / Likely | Possible + Unlikely |
|  |  | Number (%) | |
| Gastrointestinal disorders | 32 (25.6) | 26 (81.3) | 6 (18.8) |
| Musculoskeletal and connective tissue disorders | 28 (22.4) | 25 (89.3) | 3 (10.7) |
| Skin and subcutaneous disorders | 19 (15.2) | 13 (68.4) | 6 (31.6) |
| General disorders and administration site conditions | 14 (11.2) | 5 (35.7) | 9 (64.3) |
| Blood and lymphatic system disorders | 9 (7.2) | 7 (77.8) | 2 (22.2) |
| Eye disorders | 5 (4.0) | 5 (100.0) | - |
| Reproductive system and breast disorders | 5 (4.0) | 4 (80.0) | 1 (20.0) |
| Nervous system disorders | 4 (3.2) | - | 4 (100.0) |
| Investigations* | 3 (2.4) | 3 (100.0) | - |
| Vascular disorders | 1 (0.8) | - | 1 (100.0) |
| Psychiatric disorders | 1 (0.8) | - | 1 (100.0) |
| Respiratory, thoracic and mediastinal disorders | 1 (0.8) | - | 1 (100.0) |
| Renal and urinary disorders | 1 (0.8) | 1 (100.0) | - |
| Ear and labyrinth disorders | 1 (0.8) | - | 1 (100.0) |
| Other | 1 (0.8) | - | 1 (100.0) |

CTCAE = Common Terminology Criteria for Adverse Events

* 1 x ‘Weight gain’ and 2 x ‘Blood bilirubin increased’

**References Supplement 4**

1 National Cancer Institute. Common Terminology Criteria for Adverse Events (CTCAE). Available from: https://ctep.cancer.gov/protocoldevelopment/electronic_applications/docs/CTCAE_v5_Quick_Reference_5x7.pdf;

access 2024 September 10

2 World Health Organization. The use of the WHO-UMC system for standardized case causality assessment Available from: https://www.who.int/docs/default-source/medicines/pharmacovigilance/whocausality-assessment.pdf;

access 2024 September 10

**Supplement 5**

*Determination of imatinib and Nor-imatinib by tandem mass spectrometry*

Measurement of plasma IMA C_min_ and its major metabolite Nor-IMA were performed in an accredited and certified laboratory based on a protocol applying high-performance liquid chromatography (HPLC) coupled with tandem mass spectrometry (MS/MS) as described elsewhere [1, 2]. Briefly, following the thawing of 100 µl plasma, deuterated IMA-D8 and Nor-IMA-D8 (Santa Cruz Chemicals, Dallas, TX, USA) were added as internal standards. Proteins in the specimen were denatured and precipitated with zinc sulfate plus methanol and removed by centrifugation. LC-MS/MS was performed using an online SPE-HPLC-MS/MS assay with electrospray pressure ionization in the positive ion mode (API 4000, Sciex, Framingham, MA, USA). The online SPE was performed by an Oasis HLB extraction column (Waters Corporation, Milford, MA, USA), coupled to a Luna® Phenyl-hexyl column (Phenomenex, Aschaffenburg, Germany) for analytical separation. The peak areas corresponding to the m/z 494🡺394 transition for IMA and m/z 480🡺394 for Nor-IMA were measured relative to the m/z 502🡺394 reaction of the internal standard. The lower thresholds for detection were 100 ng/ml for IMA and 50 ng/ml for Nor-IMA.

**References Supplement 5**

1 Phillips RA, Ramanathan RK, Hayes MJ, Egorin MJ

Liquid chromatographic-mass spectrometric assay for quantitation of imatinib and its main metabolite (CGP 74588) in plasma.

J Chromatogr B Analyt Technol Biomed Life Sci 2003; 791:39–44

2 Klawitter J, Zhang YL, Klawitter J, Anderson N, Serkova NJ, Christians U.

Development and validation of a sensitive assay for the quantification of imatinib using LC/LC-MS/MS in human whole blood and cell culture.

Biomed Chromatogr. 2009 Dec;23(12):1251-8. doi: 10.1002/bmc.1247.

**Supplement 6**

*Analysis of the drug level and the time point of collection during treatment*

**Table S6:** Time interval after start of treatment and corresponding number of specimen collected for determination of imatinib and nor-imatinib.

| **Collection period**  **[month]** | 0-1 | 1-2 | 2-3 | 4-6 | 7-12 | 13-18 | 19-24 | 25-36 | 37-48 |
| --- | --- | --- | --- | --- | --- | --- | --- | --- | --- |
| **Number of specimen** | 20 | 28 | 25 | 50 | 38 | 19 | 27 | 23 | 18 |

**Fig. S6:** Plasma levels of imatinib (A) and nor-imatinib (B), and the resulting ratio nor-imatinib/imatiib (C) during the course of treatment. The horizontal bars denote the median.
